# Effective Sorption of Europium Ions by Interpolymer System Based on Industrial Ion-Exchanger Resins Amberlite IR120 and AB-17-8

**DOI:** 10.3390/ma14143837

**Published:** 2021-07-09

**Authors:** Talkybek Jumadilov, Khuangul Khimersen, Zamira Malimbayeva, Ruslan Kondaurov

**Affiliations:** 1 JSC “Institute of Chemical Sciences after A.B. Bekturov”, Sh. Valikhanov Str. 106, Almaty 050010, Kazakhstan; jumadilov_kz@mail.ru; 2 Institute of Natural Sciences and Geography, Abai Kazakh National Pedagogical University, Dostyk Ave. 13, Almaty 050010, Kazakhstan; huana88@mail.ru; 3 Department of Chemistry, Institute of Natural Science, Kazakh National Women’s Teacher Training University, Aiteke Bi Str. 99, Almaty 050000, Kazakhstan; malimbayeva.zamira@gmail.com

**Keywords:** interpolymer system, industrial ion exchangers, remote interaction, mutual activation, sorption, europium ions

## Abstract

The research is aimed at checking the impact of a remote interaction phenomenon on growth of sorption properties of ion-exchange resins during sorption of europium ions. Industrial ion exchangers Amberlite IR120 and AB-17-8 were selected as objects for the study. Investigation was undertaken using the following physico-chemical methods of analysis: conductometry, pH-metry, colorimetry, Fourier transform infrared (FTIR) spectroscopy, thermogravimetric analysis (TGA), and atomic emission spectroscopy. Remote interaction of the initial ion exchangers in the interpolymer system leads to their transition into highly ionized state due to formation of optimal conformation. Found that high ionization areas of Amberlite IR120 and AB-17-8 are the molar ratios Amberlite IR120:AB-17-8 = 4:2 and 1:5. The remote interaction effect provides significant increase of the following sorption properties: sorption degree, polymer chain binding degree, effective dynamic exchange capacity. A strong increase of the sorption properties (average increase for all time of remote interaction is over 50%) in the interpolymer system Amberlite IR120-AB-17-8 was observed with individual polymer structures of Amberlite IR120 and AB-17-8. The remote interaction phenomenon can be successfully used for effective modification of industrial ion exchangers for sorption of rare-earth metals.

## 1. Introduction

Nowadays, prices of rare-earth metals have significantly increased due to high demand. The global rare-earth metals (REM) market is estimated at a modest $13 billion, according to USA economics. China accounts for about 36.7% of the world’s known reserves of REM and over 70% of the world’s production of these metals [1]. Without these 17 elements that occupy the bottom of the periodic table (scandium, lutetium, thulium, etc.), the entire American high-tech industry with a turnover of trillions of dollars can stand [2]. The problem is not that some REM are really that rare in nature. They are mined much more than precious metals. But REM are obtained mainly along the way and are “brought” into laboratories using sophisticated technologies. Not surprisingly, European Union (EU) countries have created a list of 27 “critical” minerals that are important for high-tech industries and are already in short supply. Almost all REM are included. REM are used in various branches of technology: in radio electronics, instrument making, nuclear engineering, mechanical engineering, chemical industry, metallurgy [3,4,5,6,7,8].

Europium was chosen as the object of investigation due to the fact the metal is one of the most expensive rare-earth metals along with lutetium and scandium. In 2014, the price of metallic europium ranged from 800 to 2000 US dollars per kg, and europium oxide with a purity of 99.9%—about 500 dollars per kg. Europium is used in nuclear energetics as a neutron absorber in nuclear reactors. Europium oxide is used in thermochemical decomposition of water in atomic-hydrogen power engineering (europium-strontium-iodide cycle). Europium is a dopant in samarium monosulfide (thermoelectric generators), as well as an alloying component for the synthesis of diamond-like (superhard) carbon nitride. Europium monoxide, as well as an alloy of europium monoxide and samarium monoxide, are used in the form of thin films as magnetic semiconductor materials for functional electronics and, in particular, MIS (Management Information System) electronics. Europium is an indispensable component of phosphors used in cathode-ray and plasma color screens. Euro banknotes are protected from counterfeiting by phosphors based on europium. Europium tungstate is a phosphor used in microelectronics. Europium cations are used in medical diagnostics as fluorescent probes. Radioactive isotopes of europium are used in the treatment of some forms of cancer. The goal of the present work was the search of conditions of maximum sorption of europium ions from water solutions using remote interaction effect [9,10,11].

Existing sorption technologies of REM in hydrometallurgy are based on the application of ion-exchange resins [12,13,14,15,16,17,18,19]. For the study, common industrial ion exchangers were selected as sorbents: Amberlite IR120 H was chosen as cation exchanger, AB-17-8 was chosen as anion exchanger.

Amberlite IR120 H cation exchange resin is a gel type strongly acidic cation exchange resin of the sulfonated polystyrene type. It is used for water demineralisation (in H^+^ form) in co-flow regenerated units. The ion-exchange resin is a general purpose demineralization resin with a long-established track record of reliable performance in the industry. This durable resin offers a good balance of capacity and strength resulting in long lifetime for co-flow regenerated systems in industrial water treatment. The ion exchanger is an excellent general purpose cation exchange resin that can be used for a wide variety of water demineralisation applications. The operating capacity depends on several factors such as the water analysis and the level of regeneration. Amberlite IR120 H resin is suitable for industrial uses [20,21,22,23,24].

Anion exchange resin Anionite AB-17-8 is a high molecular weight polymeric substance, a monofunctional ion-exchange resin with a macroporous and gel structure. It is produced in the form of granules with a diameter of about 1 mm, from light yellow to dark yellow. By its chemical composition, it is a polymer of divinylbenzene and styrene. In aqueous solutions and water, it can enter into an exchange reaction of anion exchangers. It is used for the treatment of waste and return water. Together with the cation exchanger, they are used for water purification from weakly dissociating acids (silicic, carbonic). This substance has found wide application in the chemical industry (for purification and separation of substances), food and pharmaceutical. In this area, it is used for deep water purification. In hydrometallurgy, they are used for water treatment, namely for desalting and softening water. According to the degree of dissociation, it is classified as weakly electrolytic and strongly electrolytic, which can exchange its ions over a wide pH range. The strong base is subdivided into strong and weak acid anions. Weak electrolyte ion exchangers exchange their ions within narrow pH ranges; cation exchangers—in an alkaline environment, anion exchangers—in strongly acidic and only for strong acid anions [25,26,27,28].

The main disadvantages of the existing sorption technologies are:

1. Each ion exchanger is selective relatively to one certain ion (absence of selectivity). In other words, it cannot be used successfully for selective sorption of another metal ion;
2. After each cycle of sorption ion-exchange resins require complex process of regeneration.

**Figure 1:**
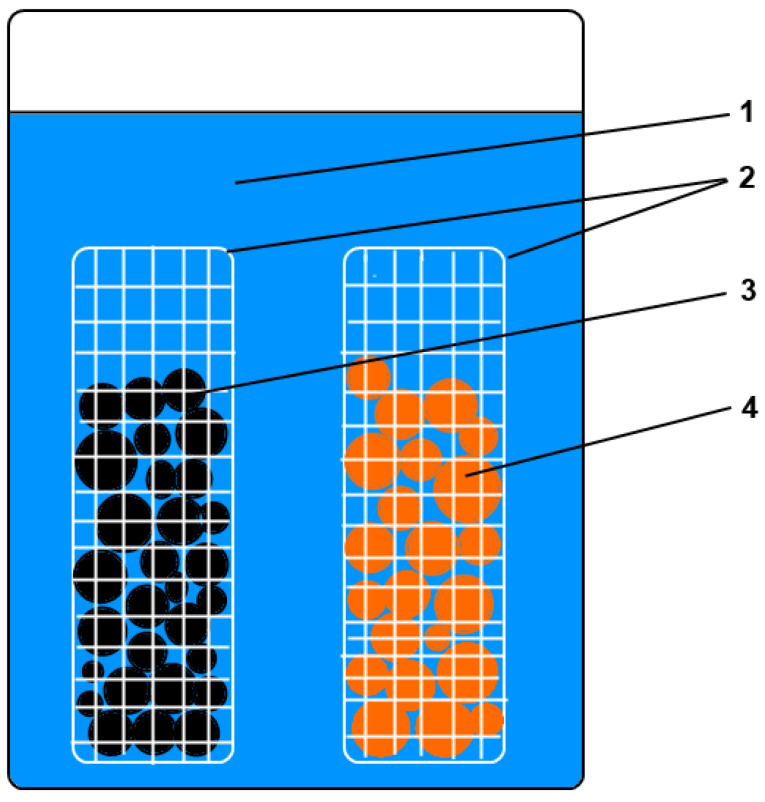
Interpolymer system based on Amberlite IR120 and AB-17-8 ion exchangers. **1**—salt solution; **2**—polypropylene cell; **3**—Amberlite IR120; **4**—AB-17-8.

Remote interaction of the macromolecules in interpolymer systems provide significant changes of initial electrochemical, volume-gravimetric and sorption properties of the components. Schematically the interpolymer system is shown in Figure 1; there are two separate ion exchangers (in our case Amberlite IR120 and AB-17-8) in the salt solution. Remote interaction phenomenon is based on dissociation of functional groups of Amberlite IR120, wherein hydrogen ions are subsequently bind by heteroatom (nitrogen atom) of AB-17-8. Due to proton balance trespassing, part of the protons are associated with the heteroatom, which in turn causes additional dissociation of undissociated functional groups of Amberlite IR120. Owing to the phenomenon of remote interaction both macromolecules are in a highly ionized state.

As mentioned above, the highly ionized state of the initial ion exchangers in interpolymer system leads to significant increase of sorption properties. Consequently, the interpolymer system Amberlite IR120-AB-17-8 will be much more effective in sorption of europium ions in comparison with individual ion exchangers Amberlite IR120 and AB-17-8.

The advantages of proposed sorption method based on remote interaction effect compared with existing sorption technologies are:

1. Possibility of fast development of several sorbents which are selective to one certain ion;
2. Possibility of creation of selective sorbents based on non-expensive industrial ion exchangers;
3. One interpolymer system can be used for selective sorption of several ions;
4. Effective separation groups of cations from anions.

These advantages of interpolymer systems over individual ion exchangers (existing sorption technologies) can be a basis for development of new sorption methods and technology in future for selective extraction of rare-earth metal ions in hydrometallurgy.

## 2. Materials and Methods

### 2.1. Materials

#### 2.1.1. Ion-Exchange Resins

**Scheme 1:**
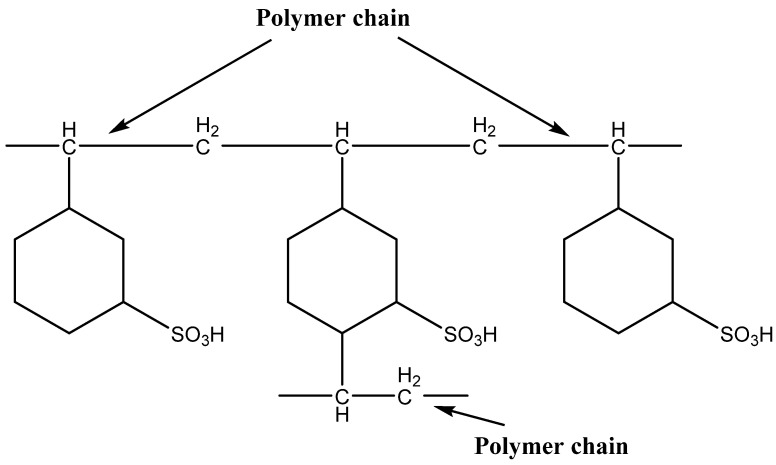
The chemical formula of Amberlite IR-120.

The formula of ion-exchange resin Amberlite IR-120 H-form is presented in Scheme 1:

The ion-exchange resin (cation exchanger) Amberlite IR-120 was synthetized by Sigma-Aldrich company (Saint-Louis, MO, USA).

**Scheme 2:**
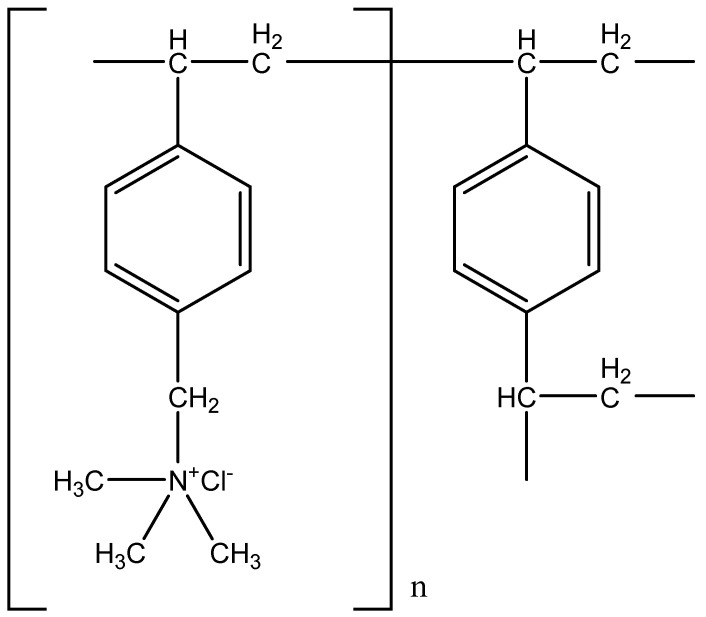
The chemical formula of AB-17-8.

The formula of ion-exchange resin AB-17-8 is presented in Scheme 2:

The ion-exchange resin AB-17-8 (anion exchanger) was synthetized by Laborfarma company (Almaty, Kazakhstan).

#### 2.1.2. Preparation of Interpolymer Systems

The Interpolymer system Amberlite IR-120-AB-17-8 is based on the phenomenon of remote interaction of macromolecules. Dispersion of each ion exchanger was crushed in analytical mill, for the experiment the particles with size 120 µm < d < 180 µm were chosen. After that the dispersion was put into special polypropylene cells with pores of 100 µm. As seen from the data provided above the pores of the cells is impermeable for the dispersion of polymers, but permeable for ions. After that, the cells are put into glass (200 mL) each in front of each other on the distance of 2 cm (Figure 1). Total amount of the dispersion (single of multiple if the interpolymer pair presents in the solution) is always constant = 6 mol (also it should be noted that sorbent mass is constant and equal 0.12 g—in case of interpolymer system it is calculated as total mass of both Amberlite IR120 and AB-17-8). For convenience of analysis of the obtained results, ratios between ion exchangers are considered as molar concentrations. With AB-17-8 share increase in interpolymer system concentration of Amberlite IR-120 decreases from 3.84 mmol/L to 0.64 mmol/L (ratios of Amberlite IR-120:AB-17-8 6:0–1:5). Concentration of AB-17-8 increases from 0.64 mmol/L to 3.84 mmol/L (ratios of Amberlite IR-120:AB-17-8 1:5–0:6) with a decrease of Amberlite IR-120 share.

#### 2.1.3. Calculation of Sorption Parameters

Europium nitrate hexahydrate (concentration 100 mg/L) was used as model salt solution.

Europium ions extraction (sorption) degree was calculated according to the formula:

$$\eta =\frac{{С}_{0}-{С}_{e}}{{С}_{0}}*100\%\;,$$

where *С*_0_—europium ions initial concentration in solution, mg/L; *С_e_*—europium ions equilibrium concentration in solution, mg/L. 

Total polymer chain binding rate was calculated according to the formula:

$$\theta =\frac{\nu_{sorbed}}{n_{1}\nu_{2}+n_{2}\nu_{2}}*100\%,$$

where *ν_sorbed_*—amount of sorbed europium ions, mole; *ν*_1_—amount of Amberlite IR120, mol; *ν*_2_—amount of AB-17-8, mol; *n*_1_, *n*_2_—amount of Amberlite IR120 and AB-17-8.

Effective dynamic exchange capacity of individual ion exchangers and interpolymer system was calculated according to the formula:

$$Q=\frac{m_{sorbed}}{m_{sorbent}},$$

where *m_sorbed_*—mass of sorbed europium ions, mg; *m*—polymer weighed portion (if two ion exchangers are present in the solution, this is calculated as summed weight of each of them), g.

### 2.2. Methods

#### 2.2.1. Measurement of Electrochemical Properties

Specific electric conductivity was investigated using Expert-002 conductometer (Econics-expert, Moscow, Russian Federation); pH was measured by 827-Metrohm pH-meter (Metrohm, Herizau, Switzerland). Measurement of electric conductivity and pH of the europium nitrate solution provides the possibility of prediction and evidence about the occurrence of remote interaction process based on the concentration of hydrogen ions in the solution.

#### 2.2.2. Measurement of Concentration

Concentration of europium ions in solution was determined by using KFK-3M spectrophotometer (Unico-Sys, Saint-Petersburg, Russian Federation) and Optima 8300 ICP-OES spectrometer (PerkinElmer, Waltham, MA, USA). All studied sorption properties (extraction degree, polymer chain binding degree, effective dynamic exchange capacity) of the interpolymer system and individual ion exchangers Amberlite IR120 and AB-17-8 are based on concentration of sorbed europium ions.

#### 2.2.3. Fourier Transform Infrared (FTIR) Spectra of Ion Exchangers Amberlite IR120 and AB-17-8

The spectrophotometer NICOLET 5700 (Thermo Fischer scientific, Waltham, MA, USA) with Fourier transmitter in area of wave numbers from 500–4000 cm^−1^ was used for obtaining Fourier transform infrared (FTIR) spectra of individual ion exchangers Amberlite IR120 and AB-17-8 and these ion-exchange structures from interpolymer system Amberlite IR120-AB-17-8.

#### 2.2.4. Thermogravimetric Analysis (TGA) of Ion Exchangers and Interpolymer System

Study of thermal properties of the ion exchangers Amberlite IR120 and AB-17-8 and the interpolymer system Amberlite IR120-AB-17-8 was made on a synchro thermogravimetric analysis/differential thermal analysis (TGA/DTA) thermoanalyzer LabSYS evo TG/DTA 1600 (Setaram, Caluire, France) in dynamic mode in temperature interval 20–630 °C with a heat rate of 10 °C/min in nitrogen atmosphere.

## 3. Results and Discussion

### 3.1. Electrochemical Properties of the Interpolymer System Amberlite IR120-AB-17-8

The remote interaction phenomenon [29,30,31,32] provides a transition of initial macromolecular structures (in the work, ion-exchange resins Amberlite IR120 and AB-17-8) into highly ionized state due to their mutual activation. Activation is a transfer of functional groups from less reactive state to more reactive state and appearance of high ionization and relaxation areas of initial ion exchangers in interpolymer pairs. Remote interaction also destroys intermolecular interchain bonds stabilized by hydrophobic fragments of divinylbenzene, which results in additional dissociation of functional groups, which are stabilized by hydrophobic fragments of divinylbenzene. The “long-range” effect in interpolymer pairs is based on two main reactions:

1. Dissociation of functional groups along with formation of water molecules while binding OH-groups;
2. Conformational changes of links of the ion exchangers stimulated by remote interaction provides destruction of interchain associates releasing functional groups with subsequent additional dissociation.

**Figure 2:**
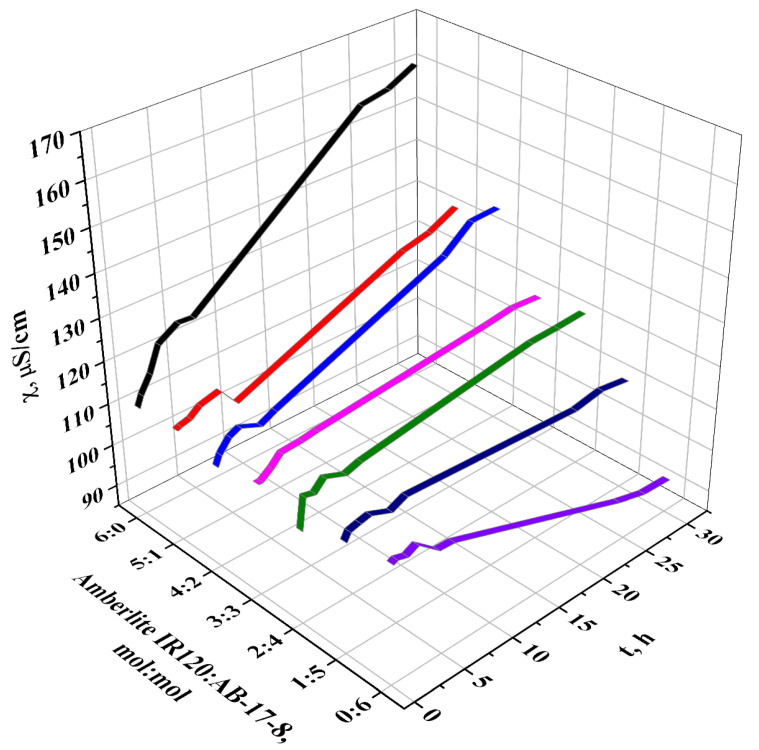
Specific electric conductivity of europium nitrate in dependence from molar ratios of ion exchangers and time.

Specific electric conductivity of europium nitrate solution in dependence from molar ratios and time is presented in Figure 2. Remote interaction of the ion exchangers leads to an increase of the electric conductivity due to the release of hydrogen ions (as known protons are one of the most mobile ions). Also it should be noted that electric conductivity increases due to the formation of hydroxyl groups in result of dissociation of polybasis. Mutual activation provides increase of ionization and relaxation areas of the initial macromolecules. Mutual activation is a transition of ion exchangers from stationary state into more reactive state. High values of electric conductivity are observed at ratios Amberlite IR-120:AB-17-8 = 5:1 and 4:2 from 24 h till 30 h of remote interaction in interpolymer system. Based only on data on specific electric conductivity it cannot be concluded exactly about high ionization areas of the initial macromolecules—for example, high values of electric conductivity could be the result of the prevalence of the functional groups dissociation process on the proton binding process.

**Figure 3:**
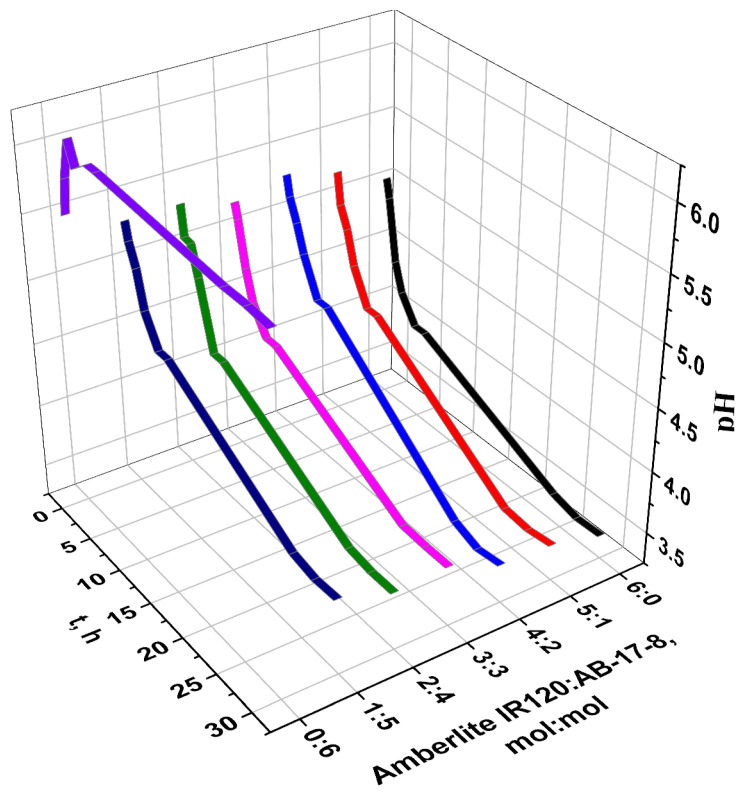
The pH of europium nitrate in dependence from molar ratios of ion exchangers and time.

The character of change of H^+^ ions amount in presence of interpolymer system Amberlite IR120-AB-17-8 is shown on Figure 3. In the presence of an individual structure, Amberlite IR120 dissociation of functional groups provides increase of content of hydrogen ions, and as a result of this pH decreases. Increase of pH values with time in presence of individual ion exchanger AB-17-8 indicates that protons from solution, which were formed due to water molecules dissociation, are bound by the heteroatom of the macromolecular structure. This strong increase of pH values points to the fact of decrease of concentration of H^+^ ions at ratios of Amberlite IR-120:AB-17-8 from 1:5 to 0:6. A decrease of concentration of protons is caused by the ionization of polybasis with the release of OH- groups which are subsequently bind with H^+^ ions forming water molecules. The obtained results point to the fact that concentration of protons increases with time for all ratios for interpolymer pairs. Such a phenomenon indicates that there is additional dissociation of functional groups of Amberlte IR120 due to binding of the released hydrogen ions. Such an additional dissociation is caused by the effect of remote interaction in interpolymer systems—the binding of protons causes decrease of their content in the salt solution due to the principle of Le Chatelier and a certain amount of undissociated groups undergoes additional dissociation. In the presence of anion exchanger AB-17-8 pH increases, which in turn points to the fact that there is binding of protons from the salt solution.

High values of specific electric conductivity point to the prevalence of functional groups dissociation over process of association of hydrogen ions. This also points to the fact of the presence of OH-groups in the solution due to dissociation of water molecules. The second process is at a limited stage here. Nevertheless, it should be noted that pH decreases with time, which in turn indicates that concentration of hydrogen ions increases. To explain this phenomenon, it can be said that there is occurrence of additional dissociation of functional groups of cation exchanger (Amberlite IR120). Additional dissociation is a consequence of the formation of a number of protons in salt solution in result of conformational changes due to this fact equilibrium is shifted to the right (release of hydrogen ions).

Based on the results obtained (Figure 2 and Figure 3), areas of high ionization of the both macromolecular structures is the molar ratios 4:2 and 1:5. Not very high values of electric conductivity at this molar ratio indicate that the overwhelming majority of released protons are bound by the anion exchanger. Due to the fact that pH values are not sufficiently low it can be concluded that ionization degree of the both macromolecules at the ratio is the highest. It is evidenced by the fact that the most of the formed hydrogen ions are associated by anion exchanger, and both methods (conductometry and pH-metry) point to a high ionization degree of both resins.

### 3.2. Sorption Properties of the Interpolymer System Amberlite IR120-AB-17-8

**Figure 4:**
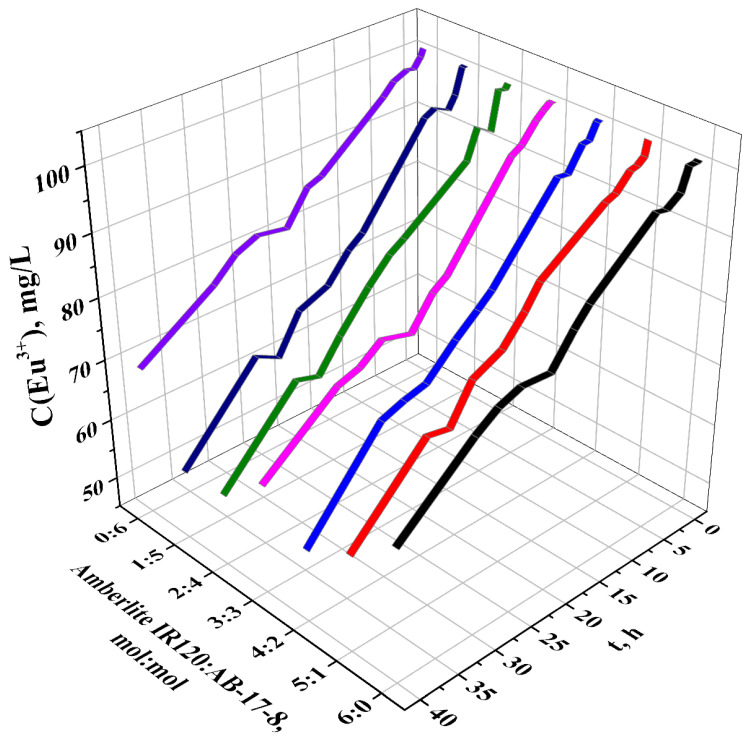
Change of concentration of Eu^3+^ during it’s sorption by interpolymer system Amberlite IR120-AB-17-8 in dependence from molar ratios and time.

Change of concentration of Eu^3+^ ions during sorption by by interpolymer system Amberlite IR120-AB-17-8 is presented on Figure 4 in dependence from molar ratios and time.

There is linear dependence of concentration change in an initial moment (0.08 h of interaction), and from 15 h exact peaks are observed at ratios Amberlite IR120-AB-17-8 4:2 and 1:5. Obtained results show that the exact tendency is observed beginning from 15 h of remote interaction—areas of high sorption are the ratios of Amberlite IR120-AB-17-8 4:2, 2:4 and 1:5. Significant sorption occurs at 27 h. After that, at 30 h relaxation effects in macromolecular structure take place, and due to this fact some part of sorbed europium is released back into the initial salt solution. Maximum sorption of europium occurs at 40 h of remote interaction of the ion exchangers at ratio 1:5, initial concentration of the rare-earth metal in the salt solution is decreased from 100.8 mg/L to 56.3 mg/L. A significant part of the metal is sorbed from the salt solution during 27 h. Over 70% of the total sorbed amount of europium is extracted at this time of interaction by individual Amberlite IR120, AB-17-8 and the interpolymer system Amberlite IR120-AB-17-8. After that as it was mentioned above—there are some relaxation effects, which result in the release of the sorbed metal back into the common solution. These data correspond to the areas of high ionization based on data on electric conductivity and pH (Figure 2 and Figure 3).

**Figure 5:**
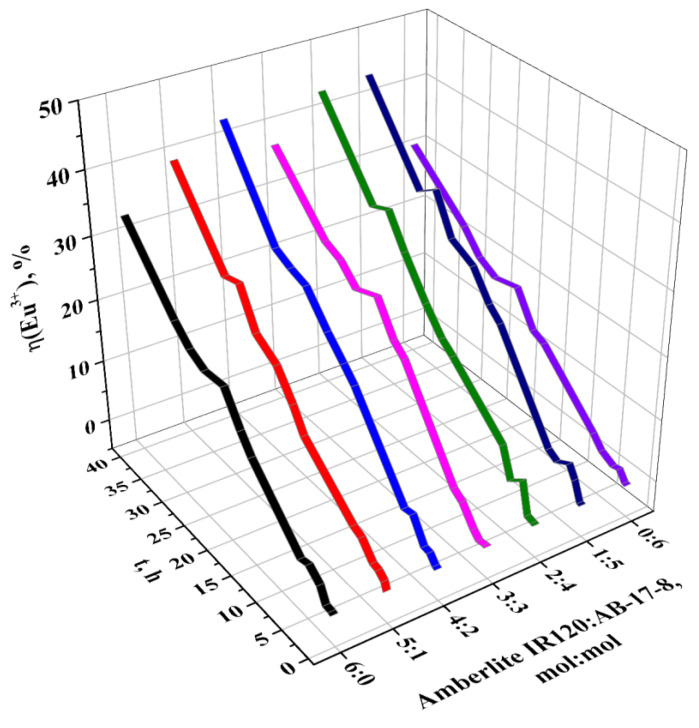
Extraction degree of Eu^3+^ ions in dependence from molar ratios and time.

The extraction degree of europium ions of the interpolymer system Amberlite IR120-AB-17-8 is presented in Figure 5 in dependence from: molar ratios and time. Figure 5 points to the fact that extraction degree increases with time. Maximum values of the parameter are observed at 40 h of interaction. A strong increase of extraction degree is observed at the following Amberlite IR120:AB-17-8 molar ratios: 4:2; 2:4 and 1:5, sorption degree is 43.07%; 43.2% and 43.7%, respectively, while the parameter is 33.17% for Amberlite IR120 and 30.64% for AB-17-8. It should be noted that the overwhelming majority of europium is sorbed during 27 h. Analyzing all the time of sorption of europium it can be concluded, that area of maximum sorption is ratio Amberlite IR120:AB-17-8 = 1:5. There is some decrease of sorption degree in an interval of time from 27 h to 30 h (sorption degree decreases from 31.90% to 30.76%), and such a phenomenon takes place due to relaxation processes in the structure of both ion exchangers over time.

**Figure 6:**
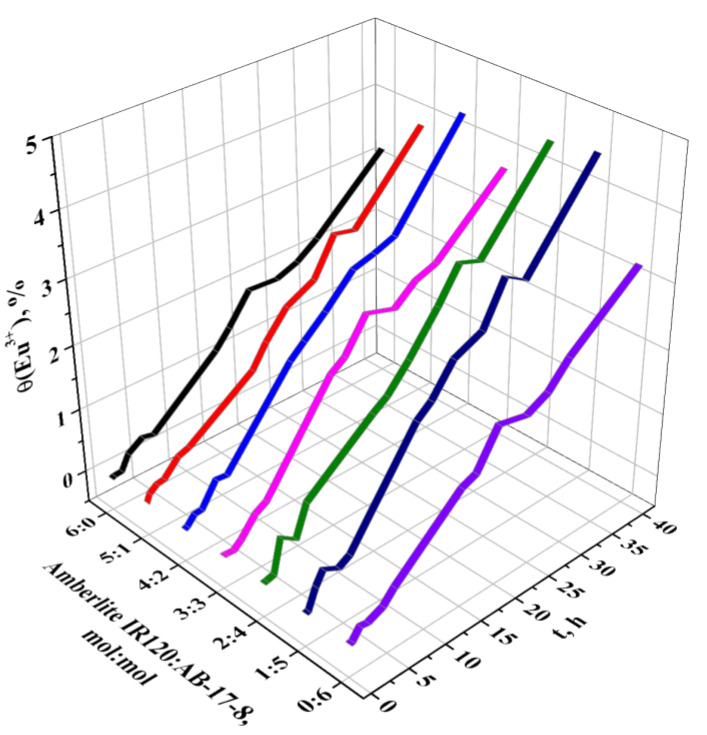
Polymer chain binding degree in dependence from molar ratios and time.

The polymer chain binding degree (relatively to europium ions) of the interpolymer system Amberlite IR120-AB-17-8 is presented in Figure 6 in dependence with molar ratios and time. The binding degree increases with time, and the most significant increase of the parameter is observed at Amberlite IR120-AB-17-8 molar ratios 2:4 and 1:5. The binding degree is 4.41% and 4.52% for these ratios at 40 h of remote interaction. Absence of “long-range” effect in case of presence of individual Amberlite IR120 and AB-17-8 leads to the fact that the binding degree is relatively low in this case: 3.21% Amberlite IR120 for and 3.22% for AB-17-8.

**Figure 7:**
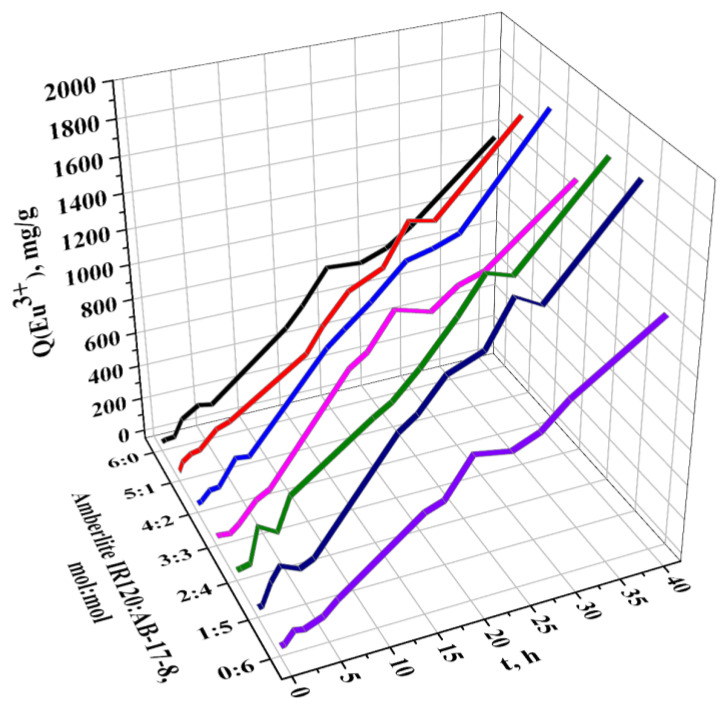
Effective dynamic exchange capacity in dependence from molar ratios and time.

The effective dynamic exchange capacity (relatively to europium ions) of the interpolymer system Amberlite IR120-AB-17-8 is presented in Figure 7 in dependence from: molar ratios and time. Individual ion exchangers do not have high values of this sorption parameter—at 40 h of interaction with the salt solution it is 1382.08 mg/g for Amberlite IR120 and 1276.67 mg/g for AB-17-8. In interpolymer pairs at molar ratios Amberlite IR120:AB-17-8 = 2:4 and 1:5 exchange capacity at 40 h is 1800.00 mg/g and 1820.83 mg/g, respectively. Mutual activation with subsequent transition of the both ion-exchange resins into a highly ionized state (it can be concluded that the ionization degree of the macromolecules is the highest at these ratios) provides significant increase of the sorption parameter.

**Figure 8:**
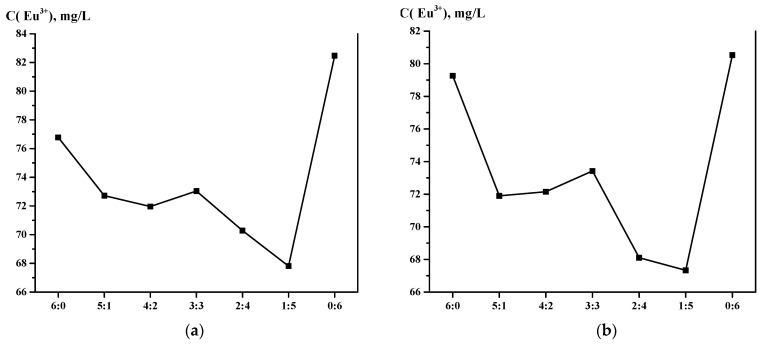
Europium ions concentration at 27 h of remote interaction of Amberlite IR120 and AB-17-8 in interpolymer system: (**a**) atomic-emission spectroscopy; (**b**) photocolorimetry.

Figure 8 represents concentration of europium ions change at 27 h of remote interaction. A clear minimum point (minimum of concentration corresponds to maximum sorption) on these curves is observed at ratio 1:5. Sorption degree for ratio Amberlite IR120:AB-17-8 = 1:5 is 32.67%, while it is 20.74% for individual Amberlite IR120 and 19.47% for individual AB-17-8.

The sorption properties (extraction degree, polymer chain binding degree, effective dynamic exchange capacity) are presented in Table 1, Table 2 and Table 3.

### 3.3. Characterisation of the Ion Exchangers Amberlite IR120 and AB-17-8 before and after Europium Ions Sorption

From the Table 1, Table 2 and Table 3 observed that maximum sorption of the europium ions in the studied interpolymer system occurs at molar ratio Amberlite IR120:AB-17-8 = 1:5, wherein the maximum increase occurs at 1.5 h. Due to this fact this molar ratio at the mentioned time was chosen for FTIR and TGA analyses.

#### 3.3.1. FTIR Spectra of the Initial Ion Exchangers and the Interpolymer Pair Amberlite IR120:AB-17-8 = 1:5

**Figure 9:**
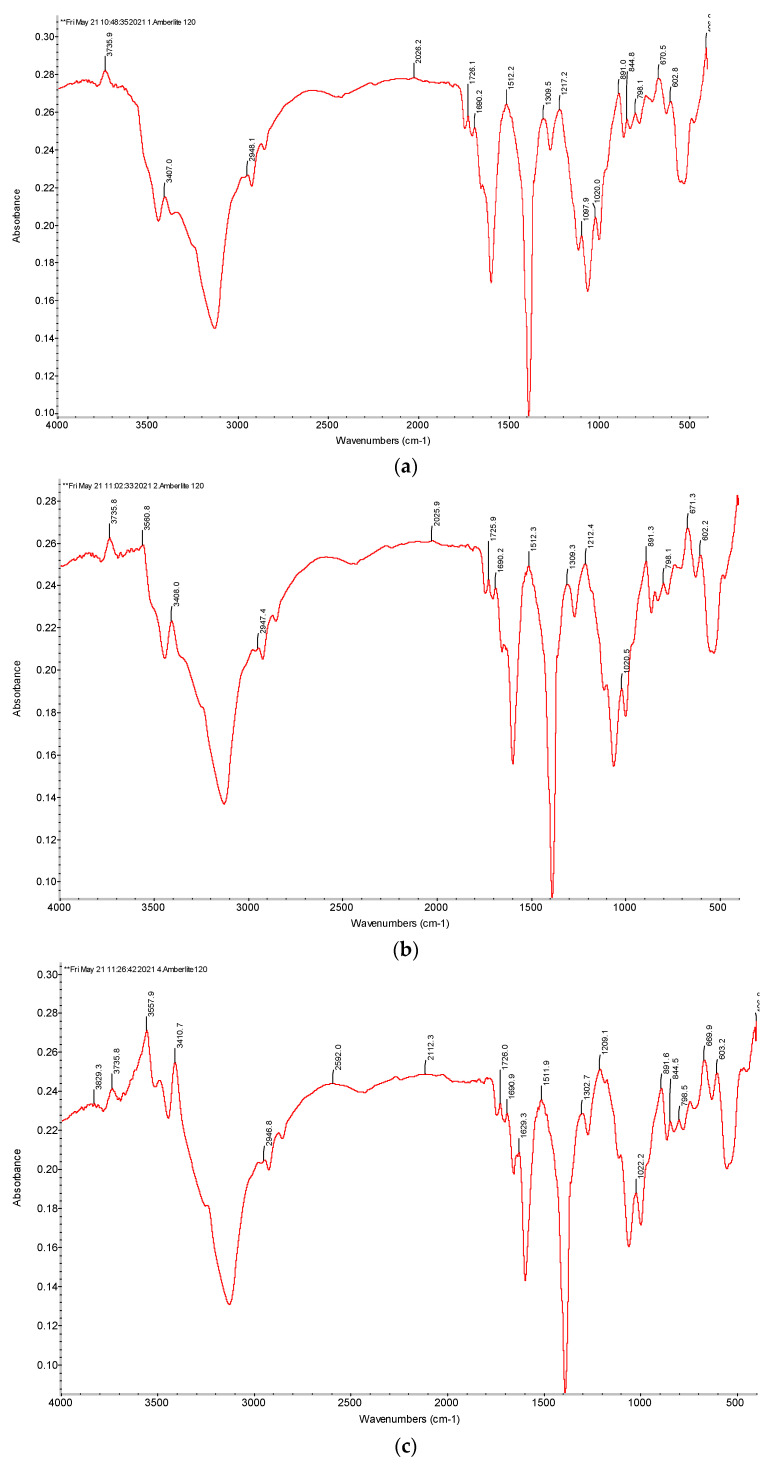
Fourier transform infrared (FTIR) spectra of (**a**) Amberlite IR120; (**b**) Amberlite IR120 6:0; (**c**) Amberlite IR120 1:5.

FTIR spectra of of initial Amberlite IR120 (without sorbed europium), individual Amberlite IR120 and Amberlite IR120 from the interpolymer pair Amberlite IR120:AB-17-8 = 1:5 from 1.5 h after the time of interaction are presented in Figure 9. Decrease of absorbance of the structures on Figure 9a–c) is a consequence of the sorption of europium ions.

**Figure 10:**
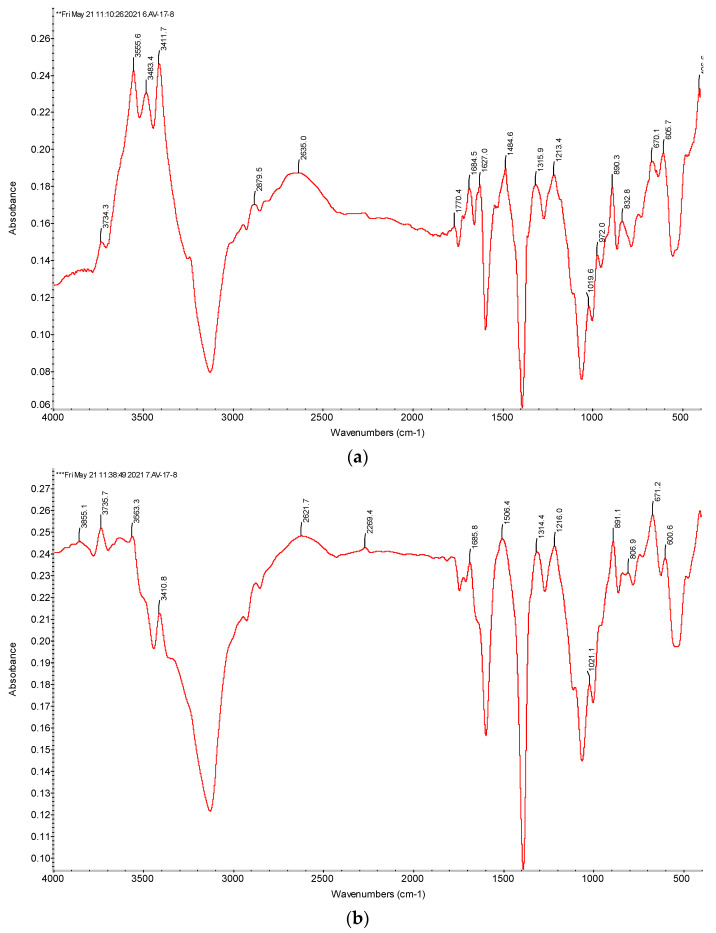

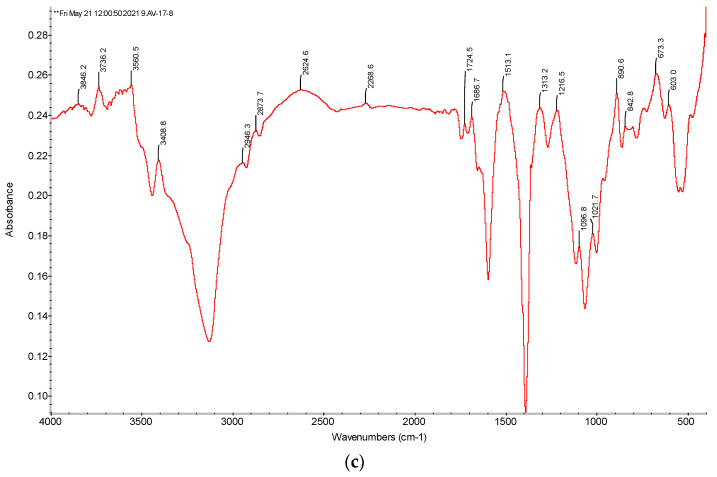
FTIR spectra (**a**) AB-17-8; (**b**) AB-17-8 0:6; (**c**) AB-17-8 1:5.

Figure 10 presents FTIR spectra of initial AB-17-8 (without sorbed europium), individual ion exchanger AB-17-8 with sorbed europium ions and AB-17-8 from ratio 1:5 with the sorbed metal ions from time of interaction 1.5 h. Ionization of the heteroatom of the ion exchanger is a reason for the increase of absorbance in Figure 10b,c due to the sorption of europium ions.

#### 3.3.2. Thermogravimetric Analysis (TGA) of Amberlite IR120; AB-17-8 and the Interpolymer Pair Amberlite IR120:AB-17-8 = 1:5

**Figure 11:**
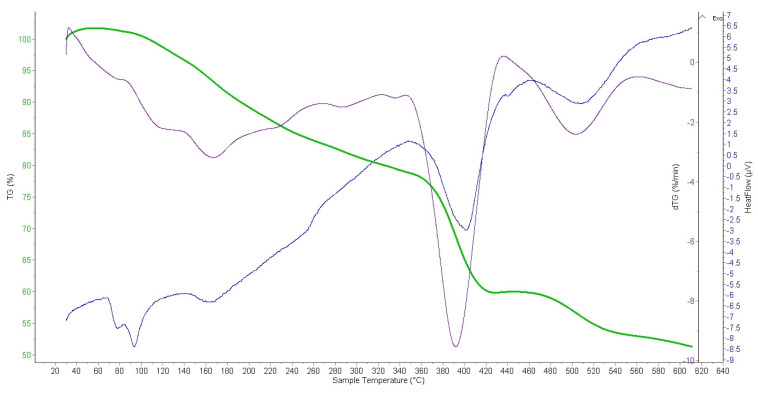
Thermogravimetric analysis (TGA) curves of europium nitrate.

As seen in Figure 11, thermal destruction of europium nitrate takes place from 350 °C to 440 °C. It is indicated by a strong decrease of mass loss (the parameter decreases from 80% to 60%), and also it should be noted that rate of mass loss increases in this temperature region.

**Figure 12:**
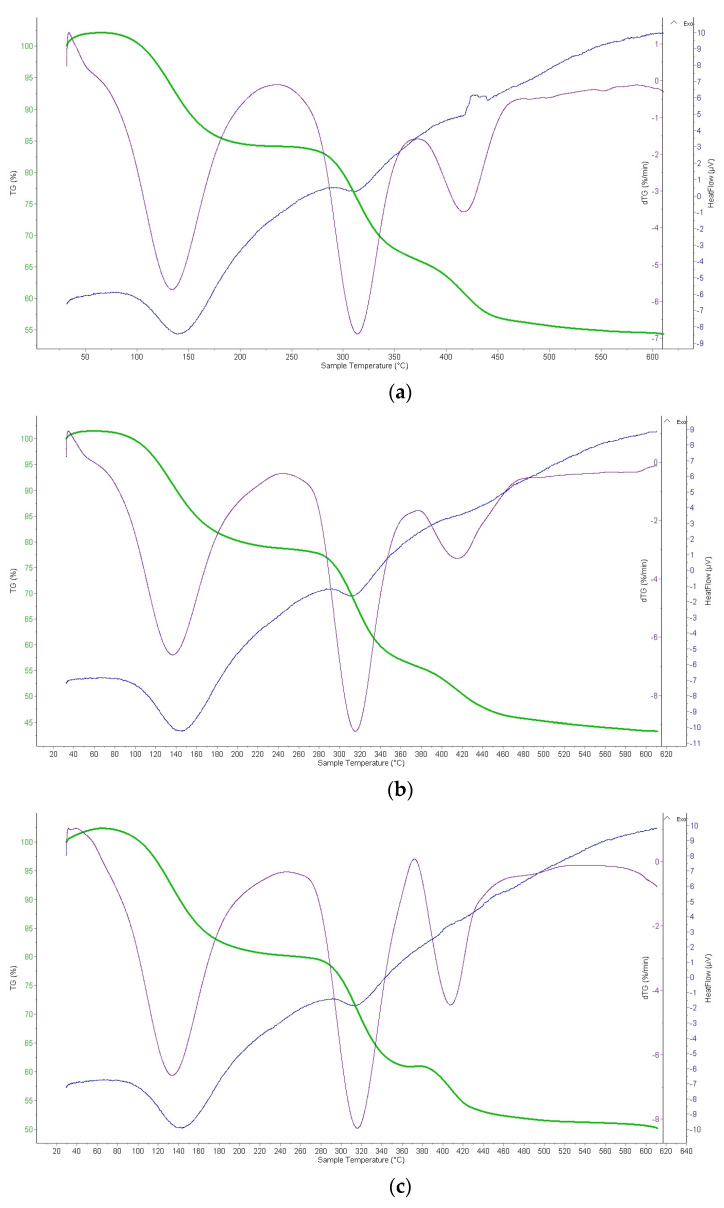
TGA curves of (**a**) Amberlite IR120; (**b**) Amberlite IR120 6:0; (**c**) Amberlite IR120 1:5.

Comparative thermal destruction of initial Amberlite IR120 (without sorbed europium), individual Amberlite IR120 and Amberlite IR120 from the interpolymer pair Amberlite IR120:AB-17-8 = 1:5 (macromolecular structures are taken 1.5 h from the time of interaction due to the fact that difference of the sorption parameters is maximum at this time in accordance with Table 4, Table 5 and Table 6) is presented on Figure 12.

**Figure 13:**
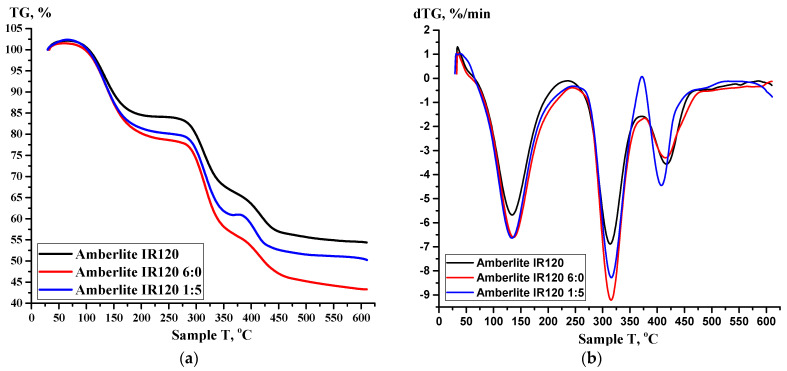
TGA curves of (**a**) mass loss and (**b**) rate of mass loss of Amberlite IR120.

Figure 13 represents comparison of thermal destruction curves (mass loss (a) and rate of mass loss (b)) of individual Amberlite IR120, Amberlite IR120 from ratio 6:0 and Amberlite IR120 from ratio 1:5. As seen from the data obtained, loss of mass of the three samples of Amberlite IR120 occurs almost simultaneously up to 125 °C. Further mass loss occurs faster for Amberlite IR120 6:0 and Amberlite IR120 1:5 due to the presence of europium ions in the polymer matrix—wherein the rate of mass loss for individual ion exchanger is higher in comparison with Amberlite IR120 from the interpolymer pair due to the fact that there is less sorbed metal in the structure. The temperature range from 400 °C to 625 °C can be named as the stabilization area with indications of the process ending.

**Figure 14:**
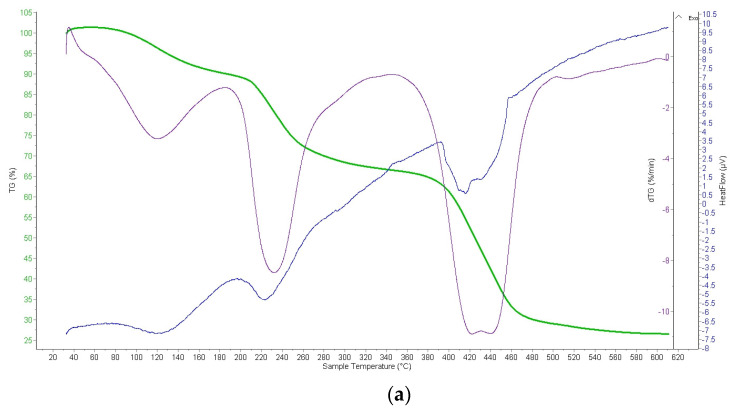

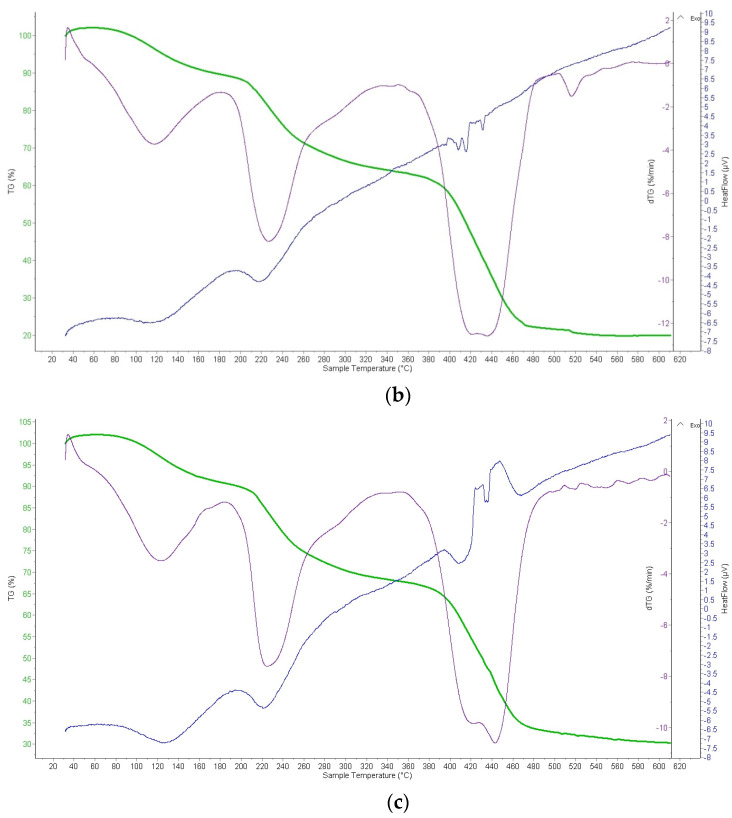
TGA curves of (**a**) AB-17-8; (**b**) AB-17-8 0:6; (**c**) AB-17-8 1:5.

Figure 14 presents TGA curves of initial AB-17-8 (without sorbed europium), individual ion exchanger AB-17-8 with sorbed europium ions and AB-17-8 from ratio 1:5 with the sorbed metal ions from time of interaction 1.5 h.

**Figure 15:**
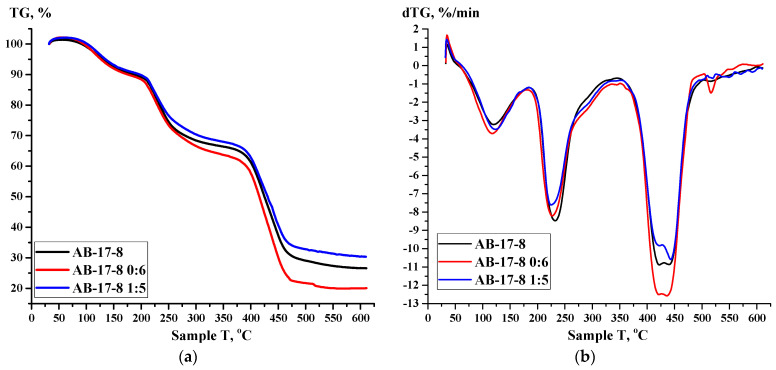
TGA curves of (**a**) mass loss and (**b**) rate of mass loss of AB-17-8.

Figure 15 represents a comparison of thermal destruction curves (mass loss (a) and rate of mass loss (b)) of individual AB-17-8, AB-17-8 from ratio 6:0 and AB-17-8 from ratio 1:5. A difference in loss of mass of the 3 samples of AB-17-8 is observed from 200 °C, before this temperature it occurs simultaneously. Strong mass loss from 375 °C to 450 °C points to thermal decomposition of the ion-exchange structure, differences in the values of the mass loss are due to sorption of europium ions by the macromolecule. The temperature area from 450 °C to 625 °C is accompanied with a slight decrease of the mass loss due to the stabilization of the thermal destruction process.

### 3.4. Growth of the Sorption Properties in Interpolymer Pair Amberlite IR120:AB-17-8 = 1:5

**Figure 16:**
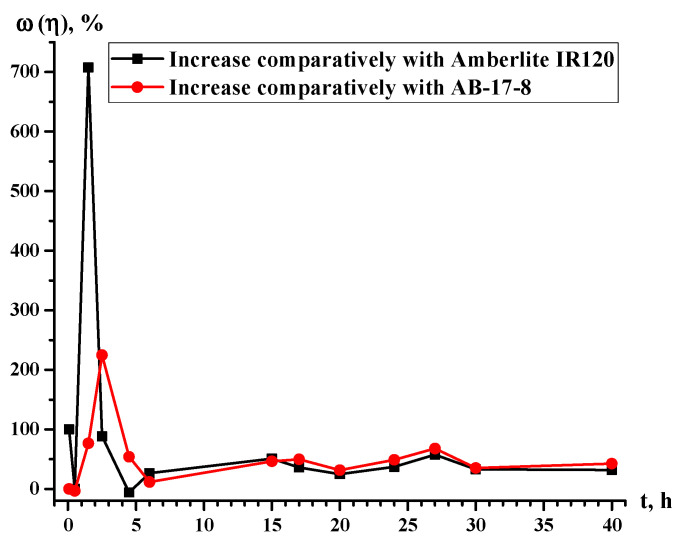
Increase of extraction degree in interpolymer pair Amberlite IR120:AB-17-8 = 1:5 relatively individual Amberlite IR120 and AB-17-8.

Growth of extraction degree of europium ions relative to the initial ion exchangers Amberlite IR120 and AB-17-8 is presented on Figure 16. The highest increase occurs during 2.5 h of remote interaction. Interpolymer pair Amberlite IR120:AB-17-8 = 1:5 has a growth of sorption degree of 707.50% at 1.5 h of interaction compared with Amberlite IR120, while the growth is 224.68% compared with AB-17-8 at 2.5 h of interaction. After that time, ionization occurs not so intensively, at 27 h growth it is 57.52% and 67.80% compared with Amberlite IR120 and AB-17-8. At 40 h growth, it is 31.75% relative to Amberlite IR120 and 42.62 relative to Amberlite IR120.

Table 4 represents growth of extraction degree of europium ions of the intergel pair compared with individual polymer structures of Amberlite IR120 and AB-17-8.

**Figure 17:**
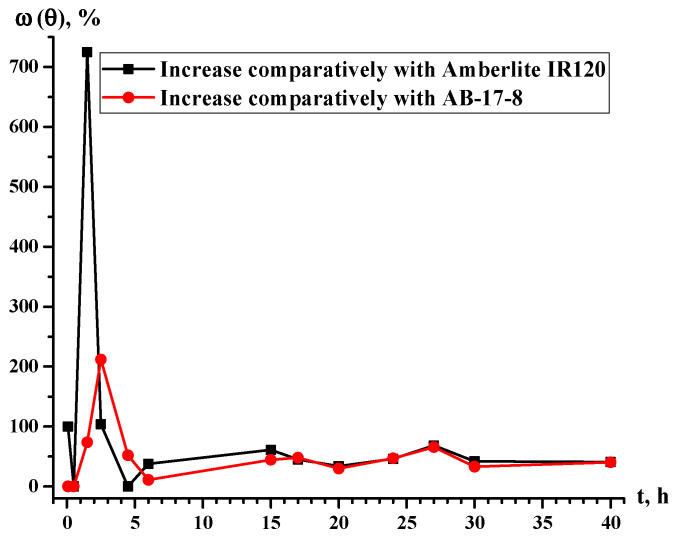
Increase of polymer chain binding degree in interpolymer pair Amberlite IR120:AB-17-8 = 1:5 relatively individual Amberlite IR120 and AB-17-8.

Growth of the polymer chain binding degree at the ratio Amberlite IR120:AB-17-8 = 1:5 occurs rather fast during 2.5 h of remote interaction (Figure 17). Binding degree increases up to 725.00% compared with individual Amberlite IR120 at 1.5 h of interaction, while the maximum growth relative to AB-17-8 (211.76%) is observed at 2.5 h. Due to relaxation effects in the structure of each ion exchanger, the ionization degree decreases with time. At 27 h of remote interaction, growth is 68.16% and 65.69% relative to individual Amberlite IR120 and AB-17-8. At 40 h growth, it is 40.81% and 40.37%, respectively.

Values of growth of polymer chain binding degree of intergel pair compared with individual ion-exchange resins presented in Table 5.

**Figure 18:**
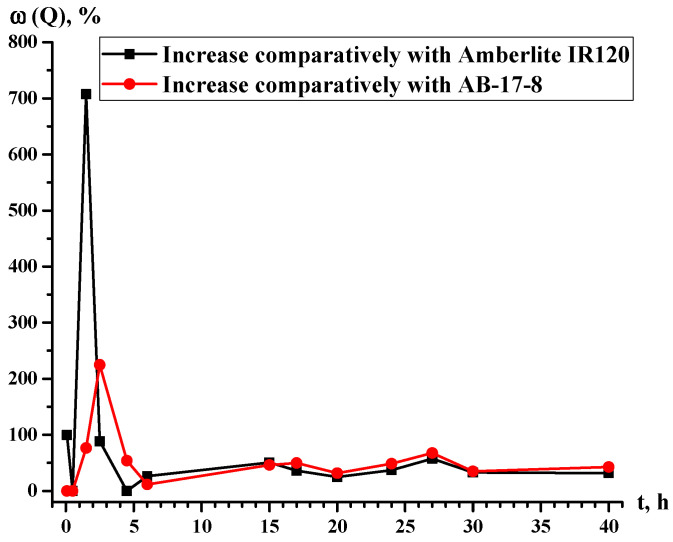
Increase of effective dynamic exchange capacity in interpolymer pair Amberlite IR120:AB-17-8 = 1:5 relative to individual Amberlite IR120 and AB-17-8.

Growth of the effective dynamic exchange capacity of the interpolymer system Amberlite IR120:AB-17-8 = 1:5 is presented in Figure 18. At 1.5 h of remote interaction, growth compared with Amberlite IR120 is 707.32%, while the maximum growth (224.70%) compared with AB-17-8 is observed at 2.5 h. Also, high growth values are observed at 27 h—57.52% and 67.80% compared with individual Amberlite IR120 and AB-17-8. At 40 h, growth is 31.75% and 42.62%, respectively.

Table 6 presents values of growth of effective dynamic exchange capacity of intergel pair compared with individual ion exchangers.

High ionization of the initial polymer structures in the interpolymer pair Amberlite IR120:AB-17-8 = 1:5 leads to formation of optimal conformation for sorption of europium ions. This is evidenced by the significant growth of sorption properties (extraction degree, polymer chain binding degree, effective dynamic exchange capacity) compared with individual ion exchangers. The strongest increase of the sorption properties relatively to Amberlite IR120 occurs at 1.5 h of remote interaction in the pair Amberlite IR120:AB-17-8 = 1:5, and it is more than 700%; compared to AB-17-8 the highest increase of the sorption properties occurs at 2.5 h of interaction, and it is more than 200%. At 27 h of remote interaction, the growth is more than 55%, while at 40 h (at the end of sorption) the growth is more than 30%.

## 4. Conclusions

Obtained data shows the possibility of prediction of remote interaction between acidic and basic ion exchangers, and their transition into highly ionized state due to mutual activation also can be predicted. The results presented above clearly demonstrate the potential of application of the remote interaction phenomenon as a new method for improvement of existing ion exchangers for maximum sorption of europium. Despite the lack of data on sorption of rare-earth metals using modern macromolecular structures, the use of interpolymer systems seems to be a relevant method for creation of principally new sorption technologies for effective extraction and rare-earth and other valuable metals. The conducted studies concerned europium, but due to the fact that all rare-earth metals are similar in chemical properties it can be predicted that the “long-range” effect will provide significantly increased sorption properties of the ion exchangers in relation to targeted rare-earth metals ions and other valuable elements.

## Figures and Tables

**Table 1:** Extraction degree of europium ions.

t. h	η (Eu^3+^), %						
	Amberlite IR120:AB-17-8 mol:mol						
	6:0	5:1	4:2	3:3	2:4	1:5	0:6
0.08	0.1	0.2	0.1	0.2	0.3	0.2	0.2
0.5	0.3	1.45	0.3	0.2	0.4	0.3	0.31
1.5	0.4	2.34	1.58	0.05	0.7	3.23	1.83
2.5	2.72	2.59	1.7	1.07	5.64	5.13	1.58
4.5	4.24	5	5.26	3.86	4.11	3.99	2.59
6	3.86	5.64	5	4.88	8.68	4.88	4.37
15	12.5	12.74	17.57	18.83	16.42	18.84	12.87
17	15.15	16.17	19.6	20.48	17.92	20.61	13.76
20	19.85	20.2	22.5	25.3	21.5	24.8	18.84
24	19.47	22.39	27	23.91	27.08	26.7	17.95
27	20.74	28.1	27.85	26.58	31.9	32.67	19.47
30	23.15	27.34	29.11	27.72	30.76	30.76	22.77
40	33.17	39.14	43.07	37.1	43.2	43.7	30.64

**Table 2:** Polymer chain binding degree.

t. h	θ (Eu^3+^), %						
	Amberlite IR120:AB-17-8 mol:mol						
	6:0	5:1	4:2	3:3	2:4	1:5	0:6
0.08	0.01	0.02	0.01	0.02	0.03	0.02	0.02
0.5	0.03	0.14	0.03	0.02	0.04	0.03	0.03
1.5	0.04	0.23	0.16	0.01	0.07	0.33	0.19
2.5	0.26	0.25	0.17	0.11	0.58	0.53	0.17
4.5	0.41	0.49	0.52	0.39	0.42	0.41	0.27
6	0.37	0.55	0.50	0.49	0.89	0.51	0.46
15	1.21	1.25	1.75	1.90	1.68	1.95	1.35
17	1.47	1.59	1.95	2.06	1.83	2.13	1.44
20	1.92	1.98	2.24	2.55	2.20	2.57	1.98
24	1.89	2.20	2.68	2.41	2.77	2.76	1.88
27	2.01	2.76	2.77	2.68	3.26	3.38	2.04
30	2.24	2.68	2.89	2.79	3.14	3.18	2.39
40	3.21	3.84	4.28	3.74	4.41	4.52	3.22

**Table 3:** Effective dynamic exchange capacity.

t. h	Q (Eu^3+^), mg/g						
	Amberlite IR120:AB-17-8 mol:mol						
	6:0	5:1	4:2	3:3	2:4	1:5	0:6
0.08	4.17	8.33	4.17	8.33	12.50	8.33	8.33
0.5	12.50	60.42	12.50	8.33	16.67	12.50	12.92
1.5	16.67	97.50	65.83	2.08	29.17	134.58	76.25
2.5	113.33	107.92	70.83	44.58	235.00	213.75	65.83
4.5	176.67	208.33	219.17	160.83	171.25	166.25	107.92
6	160.83	235.00	208.33	203.33	361.67	203.33	182.08
15	520.83	530.83	732.08	784.58	684.17	785.00	536.25
17	631.25	673.75	816.67	853.33	746.67	858.75	573.33
20	827.08	841.67	937.50	1054.17	895.83	1033.33	785.00
24	811.25	932.92	1125.00	996.25	1128.33	1112.50	747.92
27	864.17	1170.83	1160.42	1107.50	1329.17	1361.25	811.25
30	964.58	1139.17	1212.92	1155.00	1281.67	1281.67	948.75
40	1382.08	1630.83	1794.58	1545.83	1800.00	1820.83	1276.67

**Table 4:** Increase of extraction degree in interpolymer pair Amberlite IR120:AB-17-8 = 1:5 compared with individual ion exchangers.

t. h	η (Eu^3+^), %			ω (η), %	
	Amberlite IR120	Amberlite IR120-AB-17-8 = 1:5	AB-17-8	Growth Relative to Amberlite IR120	Growth Relative to AB-17-8
0.08	0.1	0.20	0.20	100.00	0.00
0.5	0.3	0.30	0.31	0.00	−3.23
1.5	0.4	3.23	1.83	707.50	76.50
2.5	2.72	5.13	1.58	88.60	224.68
4.5	4.24	3.99	2.59	−5.90	54.05
6	3.86	4.88	4.37	26.42	11.67
15	12.5	18.84	12.87	50.72	46.39
17	15.15	20.61	13.76	36.04	49.78
20	19.85	24.80	18.84	24.94	31.63
24	19.47	26.70	17.95	37.13	48.75
27	20.74	32.67	19.47	57.52	67.80
30	23.15	30.76	22.77	32.87	35.09
40	33.17	43.70	30.64	31.75	42.62

**Table 5:** Increase of polymer chain binding degree in interpolymer pair Amberlite IR120:AB-17-8 = 1:5 compared with individual ion exchangers.

t. h	θ (Eu^3+^), %			ω (θ), %	
	Amberlite IR120	Amberlite IR120-AB-17-8 = 1:5	AB-17-8	Growth Relative to Amberlite IR120	Growth Relative to AB-17-8
0.08	0.01	0.02	0.02	100.00	0.00
0.5	0.03	0.03	0.03	0.00	0.00
1.5	0.04	0.33	0.19	725.00	73.68
2.5	0.26	0.53	0.17	103.85	211.76
4.5	0.41	0.41	0.27	0.00	51.85
6	0.37	0.51	0.46	37.84	10.87
15	1.21	1.95	1.35	61.16	44.44
17	1.47	2.13	1.44	44.90	47.92
20	1.92	2.57	1.98	33.85	29.80
24	1.89	2.76	1.88	46.03	46.81
27	2.01	3.38	2.04	68.16	65.69
30	2.24	3.18	2.39	41.96	33.05
40	3.21	4.52	3.22	40.81	40.37

**Table 6:** Increase of effective dynamic exchange capacity in interpolymer pair Amberlite IR120:AB-17-8 = 1:5 compared with individual ion exchangers.

t. h	Q (Eu^3+^), mg/g			ω (Q), %	
	Amberlite IR120	Amberlite IR120-AB-17-8 = 1:5	AB-17-8	Growth Relatively to Amberlite IR120	Growth Relatively to AB-17-8
0.08	4.17	8.33	8.33	99.76	0.00
0.5	12.50	12.50	12.50	0.00	0
1.5	16.67	134.58	76.25	707.32	76.50
2.5	113.33	213.75	65.83	88.61	224.70
4.5	166.25	166.25	107.92	0	54.05
6	160.83	203.33	182.08	26.43	11.67
15	520.83	785.00	536.25	50.72	46.39
17	631.25	858.75	573.33	36.04	49.78
20	827.08	1033.33	785.00	24.94	31.63
24	811.25	1112.50	747.92	37.13	48.75
27	864.17	1361.25	811.25	57.52	67.80
30	964.58	1281.67	948.75	32.87	35.09
40	1382.08	1820.83	1276.67	31.75	42.62

## Data Availability

The raw/processed data required to reproduce these findings cannot be shared at this time due to technical or time limitation.
